# Highly pathogenic avian influenza H5N1: history, current situation, and outlook

**DOI:** 10.1128/jvi.02209-24

**Published:** 2025-03-27

**Authors:** Florian Krammer, Enikö Hermann, Angela L. Rasmussen

**Affiliations:** 1 Department of Microbiology, Icahn School of Medicine at Mount Sinai 200769, New York, New York, USA; 2 Center for Vaccine Research and Pandemic Preparedness (C-VaRPP), Icahn School of Medicine at Mount Sinai 5925, New York, New York, USA; 3 Department of Pathology, Molecular and Cell-Based Medicine, Icahn School of Medicine at Mount Sinai 5925, New York, New York, USA; 4 Ignaz Semmelweis Institute, Interuniversity Institute for Infection Research, Medical University of Vienna 27271, Vienna, Vienna, Austria; 5 Vaccine and Infectious Disease Organization, University of Saskatchewan 7235, Saskatoon, Saskatchewan, Canada; 6 Department of Biochemistry, Microbiology, and Immunology, University of Saskatchewan 7235, Saskatoon, Saskatchewan, Canada; 7 Department of Ecology and Evolution, Stony Brook University 171425, Stony Brook, New York, USA; Universiteit Gent, Merelbeke, Belgium

**Keywords:** influenza virus, avian viruses, H5N1, virus-host interactions, zoonotic infections, viral pathogenesis, pandemic risk, evolution, reassortment, influenza vaccines

## Abstract

The H5N1 avian panzootic has resulted in cross-species transmission to birds and mammals, causing outbreaks in wildlife, poultry, and US dairy cattle with a range of host-dependent pathogenic outcomes. Although no human-to-human transmission has been observed, the rising number of zoonotic human cases creates opportunities for adaptive mutation or reassortment. This Gem explores the history, evolution, virology, and epidemiology of clade 2.3.4.4b H5N1 relative to its pandemic potential. Pandemic risk reduction measures are urgently required.

## INTRODUCTION

Influenza virus pandemics are caused by influenza A viruses and happen in irregular intervals (1). The H1N1 subtype caused a pandemic in 1918 that claimed between 20 and 50 million lives, followed by H2N2 in 1957 (1–4 million deaths), H3N2 in 1968 (1–4 million deaths), and another H1N1 pandemic in 2009 (approximately 280,000 deaths) (2, 3). Typically, after causing a pandemic, these viruses establish themselves in the human population and become seasonal influenza viruses. Currently, H1N1 and H3N2 (as well as influenza B viruses) are circulating as seasonal influenza viruses in humans and cause millions of severe infections and up to 650,000 deaths per year globally (4). In addition, highly pathogenic avian influenza (HPAI) of the H5N1 subtype has caused a panzootic in birds since 2021, devastating poultry production around the world as well as infecting multiple mammalian host species. An outbreak in US dairy cattle has resulted in thousands of cows infected across 16 states, with subsequent spillback to birds and spillover to other mammals. Human H5N1 infections have concurrently increased, as exposures to infected animals have become more frequent. The risk of an H5N1 influenza pandemic continues to grow.

Pandemics are typically ignited by a zoonotic transmission event, when animal influenza viruses are transmitted across the species barrier and establish sustained transmission in the human population. While zoonotic avian influenza viruses infect humans on a regular basis, these are usually dead-end infections because the viruses are unable to transmit efficiently in humans despite their ability to cause severe disease (5–8). Replication and transmission of influenza A viruses can be inhibited in humans by restriction factors such as MxA, by the inability to interact efficiently with the host cellular machinery required for their replication, assembly, or egress, by the inability to use alpha-2,6-linked sialic acid receptors in the upper respiratory tract, and potentially many other interactions that have not yet been described (9). However, zoonotic influenza viruses can mutate to overcome these barriers, and some mutations associated with adaptation to a human host have consistently emerged during replication of these viruses in human patients (9). In addition, zoonotic influenza viruses can reassort with human (or mammalian-adapted) viruses in co-infected hosts and produce progeny viruses that can overcome host restriction and transmit well from humans to humans. In fact, the viruses that caused the 1957, 1968, and 2009 pandemics were such reassortant viruses that harbored genomic segments from human seasonal and animal influenza viruses (10). The interface between animals infected with influenza viruses and humans is a critical determinant of pandemic potential since only contact can lead to zoonosis. The bigger this interface, the greater the chance of zoonotic infections. Given the history of influenza pandemics, zoonotic H5-subtype viruses have been a concern for more than 25 years now.

## A SHORT HISTORY OF H5

H5 was recognized as a new antigenic variant of influenza virus in 1966 based on serological tests with Chicken/Scotland/59 (Smith strain) (now A/chicken/Scotland/59 [H5N1]) and Tern/South Africa/61 (now A/tern/South Africa/61 [H5N3]) (11). These viruses were classified as Hav5 (hemagglutinin avian 5) subtype which later became the H5 subtype (12). The hemagglutinin (HA) gene of A/chicken/Scotland/59 was sequenced in 1988 (13). The sequence revealed that H5 HA had a polybasic cleavage site compared to a monobasic cleavage site found in most HAs. HA needs to be activated by cleavage of HA0 into HA1 and HA2, which typically occurs extracellularly by airway or gut proteases for monobasic cleavage sites. However, polybasic cleavage sites can be cleaved intracellularly by furin-like proteases, which are expressed in all cells in the body. The presence of the polybasic cleavage motif “RKKR” in A/chicken/Scotland/59 explained earlier findings showing the virus could grow in the absence of extracellular or exogenous proteases and also linked its systemic tropism to the high pathogenicity of the virus in birds (14). In fact, both H5 HAs with monobasic cleavage sites and H5 HAs with polybasic cleavage sites exist. Viruses with monobasic H5 cleavage sites are typically not very pathogenic in chickens (low pathogenic avian influenza or LPAI) while HPAI viruses with H5 HAs with a polybasic cleavage site often kill these animals within 2–3 days (15). Of note, pathogenicity is species-specific, and some species of birds are relatively tolerant to HPAI H5N1 (e.g., mallard ducks) but can spread the virus along migration routes (16). Clinically mild infections in tolerant migratory bird species have been a major driver of outbreaks in poultry operations, as wild birds introducing HPAI into concentrated populations of farmed chickens can trigger explosive outbreaks requiring culling entire flocks.

While HPAI H5N1 was historically a significant problem for poultry farmers, it was not recognized as a human pathogen until 1997. In 1997, 18 atypical influenza virus infections in humans were reported in Hong Kong. Of these 18 individuals, 6 succumbed to infection. The causative agent was identified as an HPAI H5N1 influenza virus of the A/goose/Guangdong/1/96-lineage (17–19). When combined with other factors like mutations in one of the polymerase genes (20), the polybasic cleavage site of HA is also a virulence factor in mammals (21). However, no human-to-human transmission was observed, and experiments in ferrets also suggested inability of the virus to transmit among mammals (22). After a hiatus, HPAI H5N1 cases in humans reappeared in 2003 in Southeast Asia and began moving west via migratory birds. The virus eventually reached the Middle East (especially the Nile area in Egypt), Europe, and Africa, wreaking havoc along the way by causing die-offs of wild birds, outbreaks in poultry operations, and zoonotic spillover to humans (23). As HPAI H5N1 spread globally, the virus also evolved and diversified into many different clades and subclades based on HA sequence. This led to a complicated classification scheme for HA-based clades (e.g. clade 2.3.4.4b). Of note, while H5 was and is associated with N1 neuraminidases (NA), it can also be found associated with N2, N5, N6, N8, and other neuraminidase subtypes.

## CLADE 2.3.4.4b

**Fig 1:**
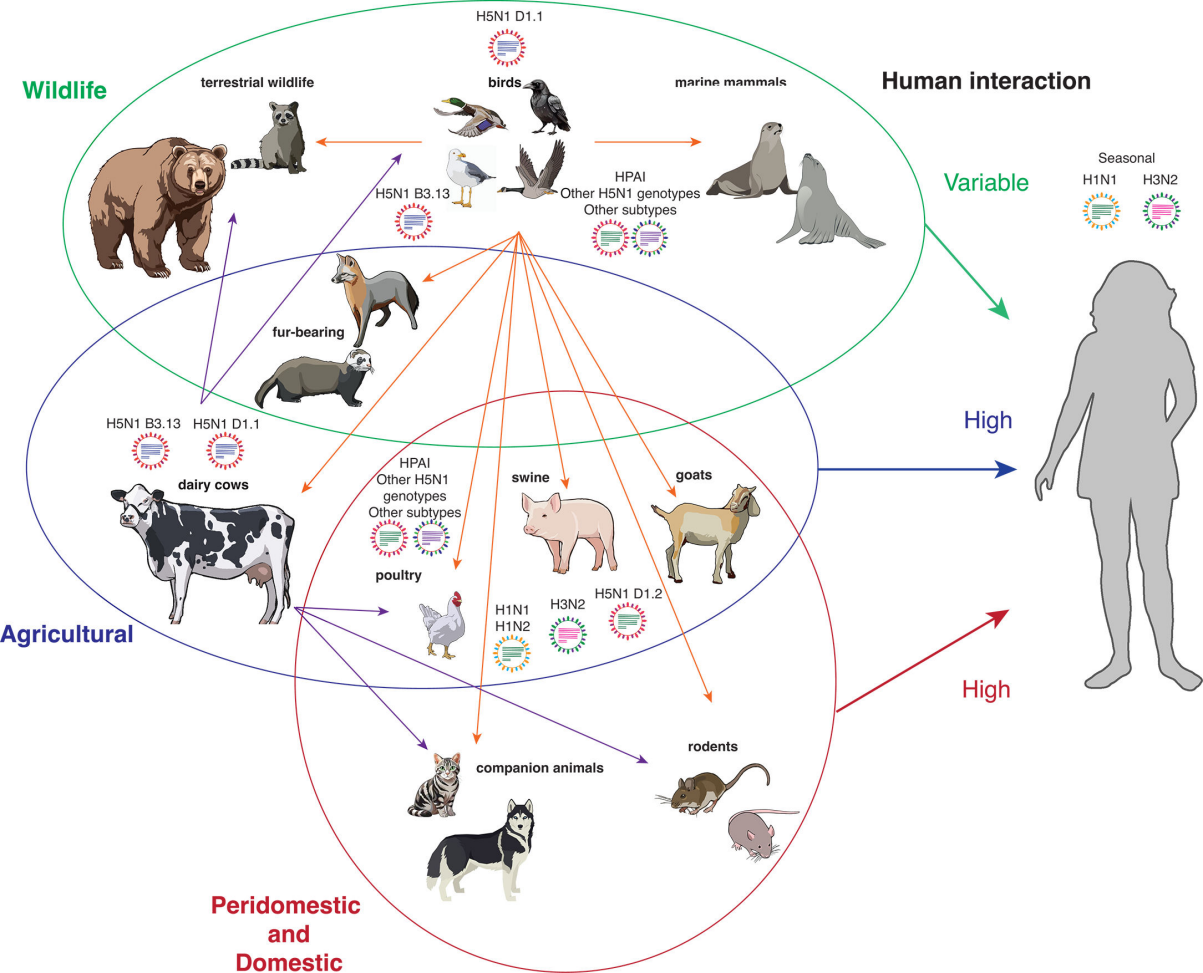
Animal species infected with H5N1. Wildlife species are encircled in green, agricultural species are encircled in blue, and peridomestic and domestic species are circled in red. Orange arrows depict transmission from an avian host. Purple arrows depict transmission from a bovine host. Viruses indicate influenza A strains known to circulate in species that present a high risk for reassortment.

In 2014 and 2015, clade 2.3.4.4 viruses expanded in Eurasia and also produced reassortant viruses like H5N2, H5N5, H5N6, and H5N8 (collectively known as H5NX), which eventually made their first incursion into the North American continent via Asia (23–26). However, in North America, they disappeared from the wild bird population within a year, and in Eurasia and Africa, they were replaced by the expanding sublineage 2.3.4.4b (23, 27). Clade 2.3.4.4b has also circulated and expanded in the form of reassortants with different NA subtypes (23). In 2020, clade 2.3.4.4b H5N8 viruses caused a surge of infection and expansion, likely originating in Egypt, into Europe and Asia. From these H5N8 strains, a new H5N1 reassortant emerged in Europe in 2021, which then expanded into Asia, Africa, and North America (23). Current clade 2.3.4.4b H5N1 viruses were first introduced into North America by migratory birds via Iceland and subsequently Atlantic Canada, but later, additional seeding occurred from Asia into the Americas as well (28–32). A hallmark of these “new” clade 2.3.4.4b H5N1 viruses is that they carry an avian N1 NA, which has a long stalk, instead of NA with a truncated stalk which is traditionally associated with HPAI H5N1 (33). Importantly, while the truncated NA stalk potentially increases pathogenicity in domestic poultry, the longer stalk seems to increase fitness in mammals and could also help the virus to expand its host range (34, 35). Furthermore, species jumps have been associated with extensive reassortment and an expansion of clade 2.3.4.4.b genotypes around the world (36). In fact, clade 2.3.4.4b viruses have been found in many different bird species (Fig. 1). In some, they show their highly pathogenic phenotype and cause mass die-offs that threaten some species of endangered birds (37–41). By now, clade 2.3.4.4b H5N1 viruses have spread to South America and Antarctica (42). They are now present on all continents except Australia, including in urban areas like New York City (43). Importantly, while HPAI historically also has infected mammalian species such as cats (44–46), this seems to be more prevalent with clade 2.3.4.4b H5N1 viruses (Fig. 1). Many species of carnivores have been infected and have died from HPAI H5N1 infection after interacting with, chewing on, or consuming infected birds, including pets (36, 47, 48) (Fig. 1). These infections are mostly dead-end infections with no onward transmission to other mammals. However, mammal-to-mammal transmission is suspected in recent clade 2.3.4.4b H5N1 outbreaks in fur farms in Spain and in Finland (49, 50). In addition, the virus seems to transmit between marine mammals in North and South America (Fig. 1), as it has caused mass die-offs that cannot be explained by exposure to birds alone (36, 51–57). Sustained transmission in mammals can select viruses with improved fitness in humans as well, which may lead to human-transmissible strains. Importantly, clade 2.3.4.4b H5N1 also reassorted in North America extensively with North American LPAI leading to a large number of different genotypes with varying pathogenicity in mammals (58–60).

## THE US DAIRY CATTLE EPIZOOTIC

In March 2024, reports came out of Texas that dairy cattle were infected with HPAI H5N1 (61) (Fig. 1). This was very unexpected since, unlike influenza C and D viruses, influenza A viruses typically do not productively infect cattle. The specific H5N1 virus that infected dairy cattle is a genotype B3.13 virus and was introduced into cows by a single bird-to-cow transmission event, most likely in Texas in late 2023 (62, 63). Although dairy farmers began to observe production losses and unexplained illness in their herds, H5N1 infection was not detected until March 2024. During the 3–4 months between spillover and detection, the virus spread rapidly through US dairy cows, likely driven by interstate transport of infected animals. This H5N1 virus has infected 973 herds in 17 states, as of 22 February 2025 (64). Although the case fatality rate in cows is low, sporadic or non-existent testing has made precise estimates of prevalence difficult to determine. The USDA did not mandate milk testing at a national scale until December 2024, so detection of infected herds and infected animals has been based on voluntary reporting and clinical symptoms or production losses. Transmission between lactating cows likely occurs through milk and milking equipment and not primarily via respiratory transmission. Milk from infected cows contains high titers (7–9 logs per milliliter) of infectious virus, which persists on milking equipment for days in the right conditions (65). Cows show distinct infection and disease phenotypes when infected by different routes. Lactating cows infected by the intramammary route develop clinical illness and have reduced or abrogated milk production, which does not recover after the acute infection has been cleared. Shedding occurs primarily through milk (66). Cows infected by the respiratory route do not develop severe illness and shed reduced amounts of virus (67, 68).

The US dairy cow outbreak has further contributed to cross-species transmission. There have been 41 cases in dairy farm workers (as of 22 February 2025 [69]). Serology studies of dairy farm workers have demonstrated that undetected human infections are occurring as well (70). Numerous animals have become infected from consuming raw milk, including cats, mice, and wild carnivores such as raccoons (61, 71). Cats frequently develop severe neurological disease and die (71). H5N1 viral RNA is now also widely detectable in commercially available milk products in the USA (72). Because pasteurization inactivates infectious virus, pasteurized milk products are safe for human consumption (73). However, pathogenicity of infections in human hosts that ingest the virus is not known, so in states that allow raw milk sales, this remains a risk to human health. Although non-human primates did not become productively infected or clinically ill following oral infection, H5N1-containing milk fed to other species of laboratory animals has led to severe disease (74, 75).

So far, it has not been possible to slow the spread of genotype B3.13 virus in dairy cattle, much less contain it. The National Milk Testing Strategy has further identified additional introductions of genotype D1.1 virus to cattle herds in Nevada and Arizona in February 2025 (64, 76). Many different genotypes of HPAI H5N1 are also still present in wild bird populations globally with no sign that circulation is slowing down ([58], this reference describes initial genotypes and provides a very useful genotyping tool). In 2024, North America observed an uptick in human cases associated with the D1.1 genotype viruses in addition to B3.13 cases, which circulate in birds and not cattle (and host a different, North American lineage N1 NA). Two of these cases have caused severe respiratory disease requiring hospitalization, with one death (77). The D1.1 genotype has also caused a number of less severe cases among poultry workers. Between both B3.13 and D1.1 genotypes of the circulating clade 2.3.4.4b viruses, the number of human cases in North America surged in 2024 and has now reached 70 in the USA (as of 22 February 2025 [69]).

## H5N1 IS DEADLY IN HUMANS, ISN’T IT?

Historically, HPAI H5N1 infections have been associated with high case fatality rates (CFRs). In the first reported human zoonotic outbreak in 1997 in Hong Kong, 6 out of 18 infected individuals died, resulting in a CFR of 33%. Between 2003 and 2023, 878 human infections with H5N1 (mostly in Southeast Asia and Egypt) have been reported. Four hundred fifty-eight of these individuals died, resulting in a CFR of 52% (78). The assumption is that large amounts of virus need to be inhaled deep into the lung to establish an infection, as H5 HA selectively binds to alpha-2,3-linked sialic acid. Because the human respiratory tract only has these receptors in the lower lungs, infection is more likely to lead to severe disease. This is also why H5N1 is thought to transmit poorly between humans, as they predominantly express alpha-2,6-linked sialic acid in the upper aiways. Mutations that increase H5 HA binding to alpha-2,6-linked sialic acid are thought to be critical for H5N1 to gain pandemic potential transmissibility. H5NX subtypes other than H5N1 seem to be less pathogenic with the exception of H5N6, which also caused 93 severe infections and 57 deaths, most of them in China (79). However, many more people may be exposed to H5N1 and develop mild or asymptomatic infections, as serology studies show. In fact, the infection fatality rate (IFR) for HPAI H5N1 viruses may be magnitudes lower than the CFR (80). So far, clade 2.3.4.4b infections have mostly resulted in mild disease, and there is much speculation why, especially when taking into account that the same type of virus causes lethal infections in many other mammals.

**Fig 2:**
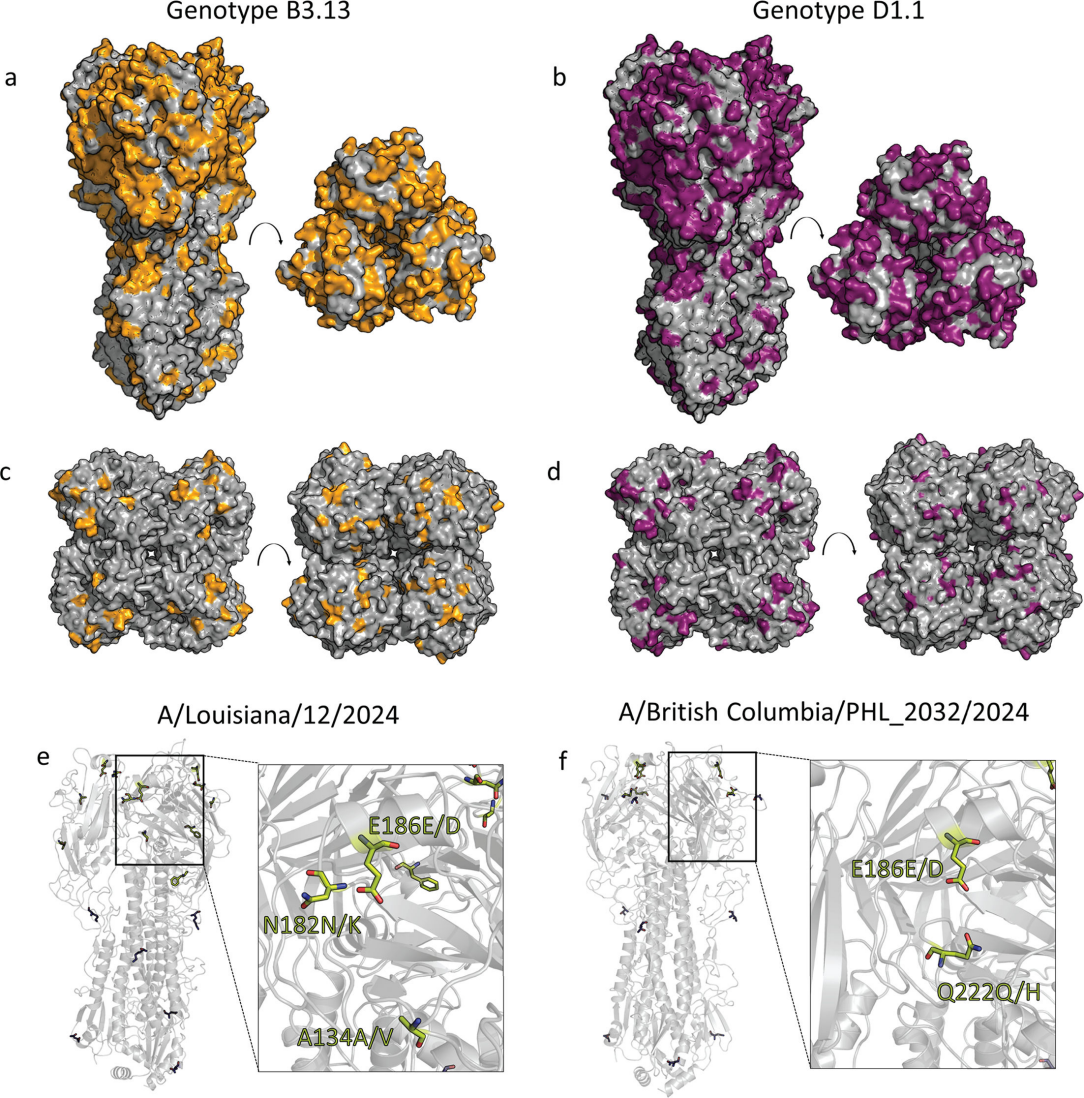
HA and NA of clade 2.3.4.4b H5N1 viruses. (a–d) Comparison of the HA and NA of a human pandemic H1N1 virus (A/California/04/2009) to genotypes B3.13 (a and c) and D1.1 (b and d) (86) of clade 2.3.4.4b H5N1. The differences in the amino acid sequence are marked in orange (B3.13) or purple (D1.1) onto H1 (a and b, PDB: 5GJS [87]) or N1 (c and d, PDB: 3NSS [88]) structures. The proteins are represented as surfaces. (e, f) Comparison of the reference genotype D1.1 (86) H5 hemagglutinin with two severe human cases from clade D1.1 (A/Louisiana/12/2024 and A/British Columbia/PHL_2032/2024). Fixed mutations are marked as blue sticks, while positions with reported mixed mutations are shown as yellow sticks (77, 89). The receptor binding site is enlarged, and mutations possibly influencing receptor binding and specificity are labeled. Models of the reference D1.1 genotype HA were created with Chai-1 (90, 91). Figures were created using PyMol version 3.0.

The underlying basis for this spectrum of pathogenicity is likely multifaceted. First, pre-existing immunity to seasonal influenza viruses, especially H1N1, significantly attenuated disease in the ferret model. Attenuation may be associated with antibody titers to the viral N1 NA and, to some degree, with T cell responses (81, 82). Cross-reactive anti-HA stalk antibodies have also been identified as correlates of protection (83) and can be found in parts of the population (see reference 84, cross-reactive titers to a clade 2.3.4.4 H5 HA are shown in Figure S1 of this reference). In fact, currently circulating seasonal H1N1 viruses possess an avian N1 NA which is related to the NA circulating in clade 2.3.4.4b H5N1 strains, and high NA inhibition titers against clade 2.3.4.4b H5N1 can be found in humans (60, 85) (Fig. 2a through d). The long stalk domain of the N1 that is prevalent in clade 2.3.4.4b viruses compared to historic HPAI H5N1 may also be an easier target for these antibodies. Of note, genotype D1.1 H5N1 viruses host a different N1 from the North American lineage, and it is unclear how well this NA is targeted by current human anti-NA antibodies.

The demographics of the human cases may also explain this. The majority of cases of both genotypes have occurred in farm workers who are generally younger adults under the age of 50 who work physically demanding jobs and are at a lower risk of severe influenza illness to begin with. Two severe human cases caused by genotype D1.1 in North America have occurred in people outside this age group, one a teenager in British Columbia, Canada, and one a man over the age of 65 in Louisiana (77) (Fig. 2e and f). A third severe human case also caused by genotype D1.1 was a woman over the age of 65 infected by backyard poultry in Wyoming and hospitalized in Colorado (92). In all these cases, the patients’ ages and pre-existing medical conditions increased disease severity risk relative to the farm workers.

Another factor may be the route of exposure. Workers in dairy farms frequently report high-risk exposures such as getting splashed in the face with milk from infected cows. Dairy exposures cause conjunctivitis more frequently than respiratory symptoms. A possible reason for this is the presence of alpha-2,3-linked sialic acid receptor on the cornea, but not the upper respiratory tract or other surrounding tissues (93). As a result of the lack of receptors in neighboring tissues, the infection is localized to the eye and is unable to spread within the host or establish a productive infection in the distant lower respiratory tract.

Finally, intensified monitoring efforts in the USA may lead to the discovery of more cases, bringing the CFR closer to the IFR. Currently, there are 70 confirmed human cases in the USA (as of 22 February 2025 [69]), the majority of which have been clinically mild and self-resolving. However, there have also been severe and fatal cases with clade 2.3.4.4b viruses. Two severe cases have been reported from Latin America after exposure to infected birds, one fatality has been reported from China, and a severe case has been reported from Vietnam (other recent and severe or fatal human H5N1 cases from Cambodia and Vietnam are associated with a different clade, clade 2.3.2.1c) (94). Three more severe cases have been reported from North America, one from Canada and two from the USA with one of the US cases being fatal. All three seem to be caused by the bird-associated D1.1 genotype rather than the cow-associated B3.13 genotype, potentially indicating differential pathogenicity in humans (77). Of note, several human H5N1 cases in the USA also had no obvious link to infected birds, cattle, or milk. At least one potential case is suspected to have been caused by consumption of raw milk from infected cows. One more case of HPAI H5N2 without a link to infected poultry was recently detected in Mexico, but this H5 virus was from the North American lineage and is unrelated to A/goose/Guangdong/1/96-like viruses (like clade 2.3.4.4b). In summary, clade 2.3.4.4b H5N1 infections in humans are happening with increasing frequency but are usually not severe compared to historic HPAI H5N1.

Nevertheless, at least two individuals died from a clade 2.3.4.4b infection, and at least five additional severe cases have been reported. Given that approximately 100 human infections with clade 2.3.4.4b H5N1 have been detected globally, this would still result in a CFR of 2% with 7% of infections being severe. A pandemic with a virus with this phenotype in humans would overwhelm healthcare systems similar to what occurred during the coronavirus 2019 (COVID-19) pandemic and cause millions more deaths. There are signs for adaptations to mammals/humans in many of the mammalian or human isolates that could lead to better replication in humans, both in terms of HA receptor recognition and those known to enhance fitness (77, 95). Mutations in HA associated with adaptation to alpha-2,6-sialic acid are particularly concerning, as they could allow for more infection and productive replication in the upper respiratory tract, which could lead to these viruses becoming transmissible in humans.

However, at least three out of the last four influenza pandemics were caused by reassortant viruses that featured both genomic segments from animal and human influenza viruses (96). Reassortment allows the virus to make rapid evolutionary leaps forward, including in terms of host adaptation. Reassortment between a human seasonal H1N1 or H3N2 virus with H5N1 clade 2.3.4.4b HPAI could result in a virus that is both pathogenic and transmissible between humans. This is perhaps the highest risk toward a future H5 pandemic. Reassortants with pandemic potential could emerge in humans or in other domestic or livestock species, such as pigs, dogs, or cats that are susceptible to both seasonal human influenza viruses and H5N1 (Fig. 1).

## COUNTERMEASURES

When severe acute respiratory syndrome coronavirus 2 (SARS-CoV-2), which was the first coronavirus to cause a pandemic in recorded history, emerged and caused COVID-19, we were entirely unprepared in terms of vaccines and antivirals. This situation is very different for influenza virus. We can access a vast body of knowledge and experience from historic influenza pandemics, seasonal influenza epidemics, and influenza virology research. Influenza virus vaccines already exist and have been used effectively for decades. H5N1 vaccines can be produced by a simple strain change to switch from seasonal influenza virus vaccines to pandemic influenza vaccines. H5N1 vaccines have been stockpiled and can be readily produced using existing facilities and established manufacturing processes and distribution networks (97). Many different H5N1 vaccines based on various candidate vaccine viruses have been produced and tested in clinical trials (98) and licensed by regulatory agencies, even though they are not available at this moment for the general public. While H5N1 vaccines are typically less immunogenic than seasonal influenza virus vaccines, they induce neutralizing antibodies when given with adjuvants in a prime-boost regimen (99–102).

Currently, of the licensed H5 vaccines, one is available with its HA matched to clade 2.3.4.4b even though its NA is of the N8 subtype and therefore mismatched (A/Astrakhan/3212/2020). This vaccine is produced by CSL Seqirus, contains MF59 as adjuvant, and is a classical split virus vaccine. Similar vaccines could, if needed, be produced by many influenza virus vaccine producers relatively quickly and licensed based on immunogenicity data from small clinical trials since the hemagglutination inhibition titer is an accepted correlate of protection for influenza virus (103).

Although the existing vaccine stockpiles are critical to a rapid and effective pandemic response, it remains insufficient to contain an emerging outbreak before it becomes a pandemic. Early in a hypothetical pandemic, even though it is likely that the H5N1 vaccine would be rolled out within 3–4 months, vaccine shortages will result from limits in production capacity, logistics, and vaccine politics. As of 22 February 2025, the US CDC has not authorized the use of the A/Astrakhan/3212/2020 H5N8 vaccines for people at high occupational risk of exposure such as dairy or poultry workers. This approach could reduce the number of human cases, protect farm workers, and lower the risk of reassortment with seasonal influenza virus strains. H5N1 vaccination has been offered to farm workers in Finland, Austria, and other European countries as a precaution. To prevent or respond to a potential H5N1 pandemic, vaccination should be deployed strategically to minimize human cases.

Alternative vaccine platforms, including recombinant HA-based vaccines, live-attenuated vaccines, and mRNA vaccines, could be employed as well. Recombinant HA-based vaccines and live-attenuated vaccines could be on the market quickly since they are currently licensed as seasonal vaccines. However, since no licensed seasonal influenza virus vaccine based on mRNA technology is available, this process could take far longer for mRNA vaccines. mRNA-based H5N1 vaccines have been tested in animal models and are also in clinical trials (results pending). They have shown to induce very high neutralizing antibody titers and induce significant protection in ferrets (104, 105). However, caution is warranted since historic H7 and H10 avian influenza virus vaccines developed by Moderna also induced extremely high titers in the ferret model but then performed poorly in humans (106, 107), suggesting a poor predictive value of animal models for protective efficacy of mRNA-based avian influenza virus vaccines.

Several classes of antivirals exist for influenza viruses. They include NA inhibitors, M2 ion channel inhibitors, and cap-snatching inhibitors that target the polymerase acidic (PA) protein. Of these, the NA inhibitors and cap-snatching inhibitors are currently in clinical use for seasonal influenza virus infections. It is likely that these drugs would also perform well for clade 2.3.4.4b H5N1 infections in the case of a pandemic (108, 109). Data from animal models currently suggest that cap-snatching inhibitors may outperform NA inhibitors (110), but no data from humans are available at present. Many promising drugs are currently also in development for influenza, including small molecules and monoclonal antibodies that could also be of value for prophylaxis or treatment of H5N1 (111). However, their use would be delayed since they are currently not licensed yet for human use.

## CONCLUSIONS AND OUTLOOK

The current situation with clade 2.3.4.4b HPAI H5N1 is concerning and presents a significant risk of an influenza pandemic. Human cases are increasing at an alarming rate, the dairy cow epizootic continues to expand in the USA, and poultry outbreaks continue sporadically but frequently. Wildlife, both avian and mammalian, is susceptible across many species to infection. The dairy cattle outbreak imperatively needs to be brought under control through vaccination of cows or better infection control or both. In regions where poultry is not vaccinated against H5N1, this should be considered to (i) protect poultry production and (ii) lower the exposure risk to humans. It would also be important to vaccinate animals in fur farms and enhance biosecurity in these operations (or, as done in Denmark and the Netherlands during COVID-19, ban the practice entirely). Some countries, including Finland and Austria, have now made H5 vaccines available to individuals with higher exposure risks such as farm workers, which is a very progressive approach that should be copied by governments globally. Unfortunately, not much can be done about virus circulation in wild bird populations (or mammals), so it is critical to protect humans and domestic animals at high risk of exposure.

There is a non-zero and increasing risk that clade 2.3.4.4b will cause the next pandemic if the virus is allowed to accumulate mammal-adaptive mutations and especially if reassortants with seasonal human influenza viruses emerge. Although pandemic risk is impossible to accurately quantify, some practices will reduce the overall risk and should be enacted at once. Reducing the virus circulating in agriculturally important species through improved epidemiological and biosecurity practices as well as vaccination will reduce opportunities for zoonotic transmission. It is essential to prevent H5N1 infections in pigs, as their co-expression of alpha-2,3-linked and alpha-2,6-linked sialic acid receptors makes them an ideal host for reassortment of both HPAI and swine or seasonal influenza viruses. Prevention and control efforts should include informing and training at-risk personnel, providing proper personal protective equipment and providing H5 vaccines to reduce human H5N1 cases. Information campaigns to inform the population of the risks of H5N1 and contact with wildlife (including in urban areas) would likely reduce the risk further. Finally, reducing the risk of human seasonal infection in at-risk personnel through offering seasonal influenza virus vaccines will limit opportunities for co-infection, mitigating the risk of reassortment.

However, it should be noted that the H5N1 situation can also be seen from another perspective. We have monitored H5N1 as a known potential pandemic threat since 1997, and it has caused human infections for more than 25 years likely with hundreds of thousands of infections over the years. Yet, H5N1 has not emerged as a pandemic virus in humans. While this is somewhat reassuring, it is also important to mention that the current clade 2.3.4.4b viruses behave (mostly undetected) infections over the years. Yet, H5N1 has not emerged as a pandemic virus in humans. While this is somewhat reassuring, it is also important to mention that the current clade 2.3.4.4b viruses behave differently than historic HPAI H5N1 viruses and that viruses in general tend to surprise us. We should not assume that H5N1 clade 2.3.4.4b viruses will not evolve into a human pathogen in the future simply because they have not in the past. An H5N1 pandemic, even with a CFR as low as 2% or lower, would result in many millions of deaths globally and devastate the global economy, akin to COVID-19. It could also disrupt food supplies, destroy ecosystems, and induce transformative social and political upheaval. Every new mammalian H5N1 infection—particularly human cases—increases pandemic risk. Controlling the ongoing H5N1 clade 2.3.4.4b is an urgent public health need.
